# Evaluating Patient Adherence to Routine and Symptom Indicated Colonoscopies During the COVID-19 Pandemic

**DOI:** 10.7759/cureus.16711

**Published:** 2021-07-29

**Authors:** Matthew C Mason, Kriti Vedhanayagam, John A Jernigan

**Affiliations:** 1 Department of Research, Alabama College of Osteopathic Medicine, Dothan, USA; 2 Department of Internal Medicine, Jackson Hospital, Montgomery, USA

**Keywords:** colorectal cancer, healthcare utilization, colon cancer screening, colonoscopy, covid-19, colonoscopy adherence rate, preventative care

## Abstract

Background and aims

The COVID-19 pandemic has led to significant changes in healthcare delivery. In response to these changes, patients have increasingly reduced healthcare utilization in several ways, such as medication compliance, cancer screenings, and routine wellness appointments. This study aims to quantify patient adherence rates to routine and symptom indicated colonoscopies during the COVID-19 and to assess patient medication compliance and utilization of healthcare facilities.

Methods

A cross-sectional study was performed at a single-center internal medicine clinic from January 2021 to April 2021. A 28-item survey was administered to patients to evaluate for adherence rates to routine and symptom indicated colonoscopies. Patients were also evaluated for rates of healthcare facility usage and medication compliance.

Results

Among 103 participants, 30.8% of patients who were due for routine colonoscopy either missed, refused, or rescheduled, while 16.7% of patients did so for symptom indicated colonoscopies. Nearly all respondents (94.2%) reported no change to medication compliance when compared to pre-COVID. A significant portion (36.9%) of patients reported missing a healthcare appointment at some point during the pandemic, and of the respondents who felt sick enough to visit the emergency department, 23.1% decided not to go.

Conclusions

During the COVID-19 pandemic, patients are deferring colorectal cancer surveillance, reducing the usage of acute care facilities, and missing routine healthcare appointments. It is important for providers to address the risks and benefits of delaying colorectal cancer screenings as well as identify physical and psychosocial barriers to patient utilization of both acute and chronic healthcare facilities. As COVID-19 restrictions inevitably continue to ease, medical providers should be aware of these potential lapses in cancer screenings and healthcare visits and be vigilant in catching patients up on their preventative health screenings.

## Introduction

Preventative health care in the form of cancer screening is an important component of primary care delivery. Definitive studies have shown that colorectal cancer screening is valuable and has improved outcomes in the average-risk population [1]. Current American College of Physicians best practice guidelines advise screening adults with average risk beginning at age 50. However, screening age varies based on individual risk and is best determined by shared decision-making between patients and providers. Routinely, patients 50 years or older with average risk make up 38% of all colonoscopy procedures [2]. If colonic lesions are identified early and removed during colonoscopy, the risk of developing colorectal cancer is decreased by up to 90 percent [3]. Clinical practice guidelines asserted by multiple organizations agree that screening followed up by treatment is successful in decreasing incidence and mortality from colorectal cancer [3, 4].

Despite the significant benefit of colorectal cancer screening, patient compliance is still an ongoing issue. Many patients lack understanding of screening purpose, underestimate the risk of colon cancer in the asymptomatic stage, or have an aversion to colorectal cancer screening modalities [5]. Patient barriers are further compounded by lower socioeconomic status, cultural barriers, and racial disparities. Lower screening rates were found among African Americans, uninsured individuals, smokers, and lower socioeconomic status populations [6].

Since December 2019, when a local epidemic of pneumonia in Wuhan, China, reached an international scale, an ensuing global pandemic wreaked havoc on healthcare systems worldwide. The causative agent was found to be a novel coronavirus (2019-nCoV) that came to be known as severe acute respiratory syndrome coronavirus 2 (SARS-CoV-2) and coronavirus disease 2019 (COVID-19) [7]. As of April 25, 2021, there were 31.8 million confirmed cases and 568,969 deaths in the United States [8]. With the rapid spread of this virus, aggressive public health measures were put in place throughout the United States and internationally to decrease disease transmission.

The impact of these restrictions, as well as the climbing number of COVID-19 cases, led to huge stressors on the healthcare system, changes in the delivery of care, and utilization of care. Increased anxiety and fear surrounding the virus have contributed to patients’ delaying, canceling, or missing routine wellness checkups and cancer screenings - including routine colonoscopies [9, 10]. While telehealth usage has grown, in-person provider visits have steeply declined by 70-80% from the baseline level [11]. Most of the decline has been seen in asymptomatic patients with a 76% reduction from average [11]. These asymptomatic patients are often the primary population that will benefit from early detection of cancer and routine screenings, such as colonoscopies.

Not only are patients refusing routine colonoscopy screenings, but also symptom indicated colonoscopies [10]. According to a review of electronic medical records in the United States during the pandemic, the average number of weekly colonoscopy screenings has decreased by 86% [12]. If this reduction in screening continues, estimates predict that there could be a precipitous drop in cancer diagnoses, leading to later initiation of treatment and worse outcomes [11]. One study showed that up to 29% of patients were non-presenters to endoscopy procedures, and of those that presented for symptom indicated endoscopies, there was a positive pathological diagnosis rate of 48.7% and a cancer diagnosis rate of 5.9% [10]. A classic representation of this delay is a case study that showed a patient with low-grade dysplasia on esophagogastroduodenoscopy (EGD) who delayed two subsequent repeat EGD’s due to COVID-19 related concerns. This patient reluctantly agreed to repeat EGD three months later and was found to have intramucosal cancer in the background of high-grade dysplasia [9]. In contrast, other studies have shown that patient willingness to undergo elective endoscopy is quite high, with rates as high as 75% and only 4% unwilling and 21% unsure [13].

While some practices have encouraged the use of other screening modalities such as fecal occult blood testing (FOBT), once screened positive, many are reluctant to undergo colonoscopy due to fear of viral transmission. Delay of endoscopy after positive screening test by more than six months carries with it a substantial risk of colorectal cancer or advanced-stage disease [14]. Statistical models have been performed to estimate the impact of delayed cancer screenings on patient morbidity and mortality. One model estimated a delay of colorectal cancer diagnosis for 18,800 patients in just a three-month period [11]. Another model, using conservative measures, estimates 33,890 excess deaths across 24 cancer types and 2,315 excess deaths due to colon cancer at a one-year period [15]. In the UK, it is estimated that over the next five years, there will be a 15.3%-16.6% increase in deaths and 25,583-27,735 years of lost life [16].

The decrease in healthcare utilization extends beyond the scope of cancer screenings and routine wellness visits. In addition to routine wellness visits, studies have shown that patients are forgoing visits to acute care facilities, particularly to the emergency department, at alarming rates [17]. Furthermore, patients are intentionally stopping, skipping, or decreasing doses of prescribed medications [18]. These factors have the potential to cause profound effects on a multitude of healthcare aspects which may include but are not limited to exacerbations of underlying autoimmune diseases, deterioration of chronic medical conditions, decreases in routine cancer surveillance, and increases in patient morbidity and mortality [16].

After a review of the current literature, there is a paucity of data that quantifies patient non-presentation to routine colonoscopy screenings, and symptom indicated colonoscopies in the United States during the COVID-19 pandemic. The current study will aim to quantify adherence to routine and symptom indicated colonoscopies in addition to healthcare utilization during the COVID-19 pandemic.

## Materials and methods

This is a cross-sectional observational study in which a 28-question survey was administered to patients at a single-center internal medicine clinic. Patients were included if they were greater than or equal to the age of 19 years old and were under the care of a physician at the single-center internal medicine clinic. Patients were excluded if they were under the age of 19 or were unable to answer or comprehend the administered survey. There were 126 completed surveys, and 23 were excluded due to missing or incomplete responses leaving 103 eligible surveys for data analysis. The survey consisted of a series of questions which first asked participants for demographic information (see Table 1) that included age, sex, race/ethnicity, education, household size, marital status, insurance type, history of colonic polyps, history of psychiatric disorders, prior COVID-19 infection, and underlying medical conditions. Additionally, there were questions about patient compliance regarding medication use, healthcare appointments, routine colonoscopies, symptom indicated colonoscopies, and visits to the emergency department. Patients who refused, rescheduled, or missed a colonoscopy were asked the reason why they missed and their plans to reschedule. Statistical analysis was performed using Statistical Package for the Social Sciences (SPSS) software version 1.0.0.1058 (IBM Corp, Armonk, New York). This study has been reviewed and approved for ethical standards by the Institutional Review Board (HS201215-EX). 

**Table 1:** Demographic data SD = Standard deviation; GED = General education development

Characteristic	Data (%)
Age	
Mean (SD)	64.5 (10.1)
Range	41-90
Sex	
Male	35 (34.0)
Female	68 (66.0)
Race	
African American/Black	97 (94.2)
White	5 (4.9)
Other	1 (1.0)
Education	
Less than high school degree	1 (1.0)
High school degree or equivalent (GED)	13 (12.6)
Some college but no degree	20 (19.4)
Associate's degree	7 (6.8)
Bachelor’s degree	20 (19.4)
Graduate degree	42 (40.8)
Household size	
1	26 (25.2)
2	53 (51.5)
3	12 (11.7)
4	10 (9.7)
5	1 (1.0)
6	1 (1.0)
Marital status	
Single	14 (13.6)
Married	58 (56.3)
Divorced	15 (14.6)
Widowed	16 (15.5)
Health insurance type	
Private	54 (52.4)
Medicare	35 (34.0)
Medicaid	1 (1.0)
Medicare with supplement	9 (8.7)
Medicare & Medicaid	4 (3.9)
History of colonic polyps	
Yes	36 (35.0)
No	67 (65.0)
History of psychiatric condition	
Yes	19 (18.4)
No	84 (81.6)
Prior COVID-19 infection	
Yes	14 (13.6)
No	89 (86.4)
Underlying medical conditions	
Hypertension	82 (79.6)
Diabetes	40 (38.8)
High cholesterol	53 (51.5)
Cardiovascular disease	11 (10.7)
Chronic lung disease	5 (4.9)
Chronic kidney disease	1 (1.0)
None	13 (12.6)
Number of underlying medical conditions	
0	13 (12.6)
1	31 (30.1)
2	25 (24.3)
3	26 (25.2)
4	7 (6.8)
5	1 (1.0)

## Results

The mean age was 64.5 (SD = 10.1), with the youngest patient being 41 years old and the oldest being 90 years old. The majority of respondents were female (66%), whereas male respondents comprised 34% of the cohort. The study was primarily composed of African American patients at 94.2% of the study population. This study had a variety of education levels, with the majority of patients having received some form of a college degree, including an associate’s degree, bachelor’s degree, or graduate-level degree. 

Hypertension was the most common condition present in 79.6% of patients, while high cholesterol (51.5%) was listed as the second most common underlying condition. Diabetes (n=40, 38.8%) was quite prevalent while chronic lung conditions (n=5, 4.9%) and chronic kidney disease (n=1, 1%) were uncommon in this study. In this cohort, underlying conditions were almost ubiquitous, with 87.4% of respondents who reported at least one underlying medical condition and 57.3% who reported at least two underlying conditions. There was a substantial proportion of patients with a history of colonic polyps (n=36, 35%). An underlying psychiatric condition (18.4%) was not uncommon. 

Medication compliance was quite high, with 94.2% of patients reporting that they were taking their prescribed medications the same as before the pandemic, while only 4.9% of patients reported decreasing or skipping doses (see Table 2). Among the 12.6% of the study who personally felt the need to visit the hospital emergency department, 23.1% of them did not go. There were 26 (25.2%) patients who were due for a routine colonoscopy, and 30.8% of them refused, rescheduled, or missed their routine colonoscopy. The majority of the patients who missed their colonoscopy reported that it was due to either a fear of contracting COVID-19 (37.5%) or because the hospital or endoscopy center was not performing routine colonoscopies (37.5.%). In this cohort, only six (5.8%) respondents were advised to undergo a symptom indicated colonoscopy, of which one (16.7%) refused, rescheduled, or missed for fear of contracting COVID-19. 

 

**Table 2:** Survey results of patient compliance

Survey question	Data (%)
Patient canceled, rescheduled, or missed healthcare appointment	
Yes	38 (36.9)
No	65 (63.1)
Medication compliance	
Yes, the same as before pandemic	97 (94.2)
Decreased or skipped doses	5 (4.9)
Do not take any medications	1 (1.0)
Patient felt the need to go to an emergency room	
Yes	13 (12.6)
No	90 (87.4)
If patient felt the need to go, did they go?	
Yes	10 (76.9)
No	3 (23.1)
Reason for not going to an emergency room	
Went to another healthcare facility	1 (33.3)
Other	2 (67.7)
Patients due for a routine colonoscopy	26 (25.2)
Patients who refused, rescheduled, or missed routine colonoscopy	8 (30.8)
Patients who agreed to undergo routine colonoscopy	18 (69.2)
Reason for missing routine colonoscopy	
Fear of contracting COVID-19	3 (37.5)
Hospital/office was not performing routine colonoscopies	3 (37.5)
Other	2 (25.0)
Plans to reschedule missed routine colonoscopy	
<6 months	3 (37.5)
>6 months	1 (12.5)
Reschedule when I receive COVID-19 vaccine	2 (25.0)
Will not reschedule	1 (12.5)
Other	1 (12.5)
Patients advised to undergo symptom indicated colonoscopy	6 (5.8)
Patients who refused, rescheduled, or missed symptom indicated colonoscopy	1 (16.7)
Patients who agreed to undergo symptom indicated colonoscopy	5 (83.3)
Reason for missing symptom indicated colonoscopy	
Fear of contracting COVID-19	1 (100)
Plans to reschedule symptom indicated colonoscopy	
<6 months	1 (100)
Hypothetically, patient would undergo routine colonoscopy	
Yes	65 (63.1)
No	9 (8.7)
Unsure	29 (28.2)
Hypothetically, patient would undergo symptom indicated colonoscopy	
Yes	79 (76.7)
No	6 (5.8)
Unsure	18 (17.5)

**Figure 1:**
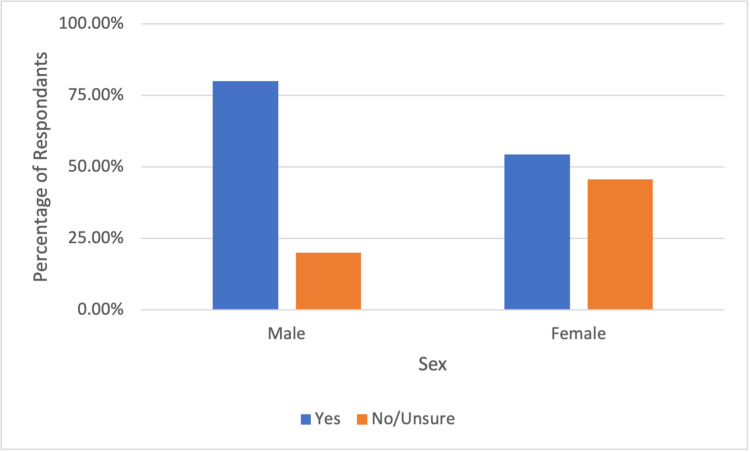
Male and female respondent willingness to undergo routine colonoscopy

**Figure 2:**
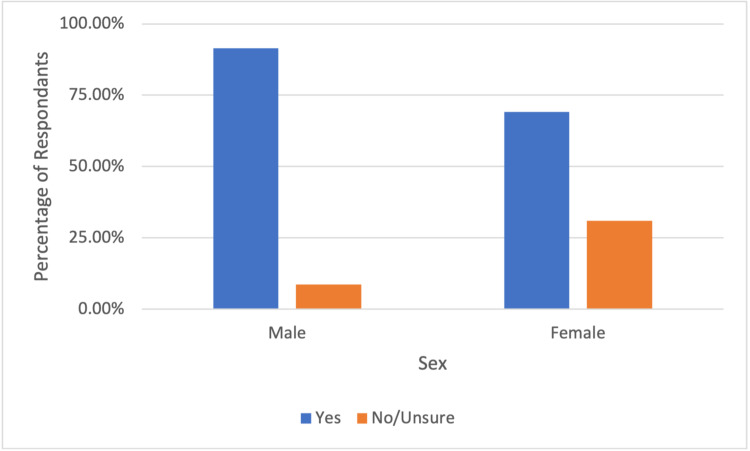
Male and female respondent willingness to undergo symptom indicated colonoscopy

When asked if the patient would hypothetically undergo a routine colonoscopy, most patients (63.1%) said “yes,” while a larger percentage (76.7%) of patients would agree to a symptom-indicated colonoscopy. In this study, 36.9% and 23.3% of patients said “no or unsure” to undergoing a hypothetical routine colonoscopy and a symptom indicated colonoscopy, respectively. In subgroup analysis, females had lower rates of willingness to undergo a routine colonoscopy and symptom indicated colonoscopy at 54.4% and 69.1%, respectively, when compared to their male counterparts at 80% and 91.4% (see Figure 1 and Figure 2).

## Discussion

This cross-sectional study demonstrated a significant decline in compliance with both routine and symptom indicated colonoscopies. Our study shows that the rate of missing a routine colonoscopy was 30.8%, and the rate of missing a symptom-indicated colonoscopy was 16.7%. Our findings surrounding those with non-symptomatic routine colonoscopies align with data showing that there has been a decline in asymptomatic patients seeking routine screening during the COVID-19 pandemic. Fear of COVID-19 and the consequences of contracting this virus have overshadowed primary healthcare concerns. Patient attitudes have shifted away from long-term impacts and focused on the short-term consequences of interacting with the healthcare system during the global pandemic.

Isolating public health safety measures and mounting fear of severe illness has led to deleterious effects on patients’ mental health. Many studies have reported increased levels of stress, anxiety, and depression during the pandemic [19-21]. In one meta-analysis, the prevalence of stress was 29.6%, and the prevalence of anxiety and depression was 31.9% [22]. Individual risk factors such as preexisting mental health conditions, chronic health issues, and social restriction and isolation add to the psychological impact of the global pandemic. We believe that these adverse psychological responses to the stressors of the global pandemic have contributed to patient non-presentation at appointments. Our study also shows that a larger number of females were unwilling or unsure about undergoing a routine colonoscopy when compared to their male counterparts. This is in concordance with studies showing that the female gender is strongly associated with more negative impacts due to COVID-19 stressors [19]. 

Mounting fear about COVID-19’s high mortality rate, contagiousness, and restricting public health policies have played a role in noncompliance. Despite the prevalence of underlying chronic health issues in our patient population, a large proportion (36.9%) missed, rescheduled, or canceled a healthcare appointment. Among those who thought they might need emergency care, 23.1% did not seek this care due to fear of accessing healthcare resources in the setting of the pandemic. However, contrary to a prior study indicating that many patients were no longer taking medications as directed, our patient population reported a high rate of compliance with prescribed medications [18]. Prior to the pandemic, chronic health conditions, such as diabetes, were linked to higher rates of psychological distress when compared to the general population [19]. With well-documented increases in the severity of COVID-19 disease progression with comorbidities such as diabetes mellitus, it is evident that concern over contracting the virus is enough to dissuade those with underlying health issues from attending routine appointments [23]. These findings are consistent with previous data that shows patient visits to clinics during the pandemic have decreased by 76% in asymptomatic patients and 73% in patients with chronic conditions [10]. 

By better understanding patient adherence during this dynamic global pandemic, physicians and medical providers will be better equipped to counsel patients on routine and symptom indicated colorectal cancer screenings. In addition, physicians and medical providers will be able to make further adjustments to their patient care strategies to improve patient compliance and healthcare utilization and, thus, decrease morbidity and mortality.

This study has several limitations. While this study is one of the first in the United States with emerging data exploring the shift in patient attitudes towards routine and symptom indicated colonoscopies during the global COVID-19 pandemic, it is limited by the small sample size and its observational nature. The small sampling size skewed towards one demographic may also contribute to confounding variables and would limit its generalization to the population. While not directly measured in this study, our respondent population was recruited from an area where household income and academic metrics consistently trail national averages [24, 25]. In this particular socioeconomic demographic, studies have shown decreased utilization of healthcare resources, and therefore, our study participants may inherently have lower levels of adherence to routine screenings, medication use, and healthcare utilization [26, 27].

## Conclusions

This study demonstrates that in response to the COVID-19 pandemic, patients are missing colonoscopy screenings at a high frequency. In addition to cancer surveillance, patients are also utilizing healthcare facilities at lower rates than before the pandemic. The decline in routine health screenings could lead to devastating outcomes in the future, especially for those with chronic health issues who benefit most from routine care. Therefore, we hope these findings will help providers better counsel patients about the risks and benefits of colorectal cancer screening and address barriers to healthcare delivery in the setting of the ongoing pandemic.
